# Analysis of the primary sources of quantitative adult plant resistance to stripe rust in U.S. soft red winter wheat germplasm

**DOI:** 10.1002/tpg2.20082

**Published:** 2021-02-17

**Authors:** Brian P. Ward, Keith Merrill, Peter Bulli, Mike Pumphrey, Richard Esten Mason, Mohamed Mergoum, Jerry Johnson, Suraj Sapkota, Benjamin Lopez, David Marshall, Gina Brown‐Guedira

**Affiliations:** ^1^ USDA Agricultural Research Service Plant Science Research Unit Raleigh NC 27607 USA; ^2^ Current Address: Department of Horticulture and Crop Science The Ohio State University Wooster OH 44691 USA; ^3^ Department of Crop and Soil Sciences North Carolina State University Raleigh NC 27607 USA; ^4^ Department of Crop and Soil Sciences Washington State University Pullman WA 99164 USA; ^5^ Department of Crop, Soil and Environmental Sciences University of Arkansas Fayetteville AR 72701 USA; ^6^ Current Address: Department of Soil and Crop Sciences Colorado State University Fort Collins CO 80523 USA; ^7^ Department of Crop and Soil Sciences and Institute of Plant Breeding, Genetics & Genomics University of Georgia Griffin GA 30223 USA

## Abstract

Stripe rust, or yellow rust (*Puccinia striiformis* Westend. f. sp. *tritic*), is a disease of wheat (*Triticum aestivum* L.) historically causing significant economic losses in cooler growing regions. Novel isolates of stripe rust with increased tolerance for high temperatures were detected in the United States circa 2000. This increased heat tolerance puts geographic regions, such as the soft red winter wheat (SRWW) growing region of the southeastern United States, at greater risk of stripe rust induced losses. In order to identify sources of stripe rust resistance in contemporary germplasm, we conducted genome‐wide association (GWA) studies on stripe rust severity measured in two panels. The first consisted of 273 older varieties, landraces, and some modern elite breeding lines and was evaluated in environments in the U.S. Pacific Northwest and the southeastern United States. The second panel consisted of 588 modern, elite SRWW breeding lines and was evaluated in four environments in Arkansas and Georgia. The analyses identified three major resistance loci on chromosomes: 2AS (presumably the 2NS:2AS alien introgression from *Aegilops ventricosa* Tausch; syn. *Ae. caudata* L.), 3BS, and 4BL. The 4BL locus explained a greater portion of variance in resistance than either the 2AS or 3BS loci in southeastern environments. However, its effects were unstable across different environments and sets of germplasm, possibly a result of its involvement in epistatic interactions. Relatively few lines carry resistance alleles at all three loci, suggesting that there is a pre‐existing reservoir of enhanced stripe rust resistance that may be further exploited by regional breeding programs.

## Core Ideas

- Southeastern U.S. winter wheat germplasm contains multiple stripe rust resistance loci
- A QTL located on chromosome 4B is important for stripe rust resistance in the Southeast
- The *Yr17* resistance gene on chromosome 2A has been defeated in the southeast for several years
- A *Yr17*‐linked adult plant resistance gene still offers quantitative stripe rust resistance
- The 4B resistance QTL may have unstable effects across environments and sets of germplasm

**Abbreviations**
APR: adult‐plant resistance
ASR: all‐stage resistance
BLUE: best linear unbiased estimator
GS: growth stages
GWA: genome‐wide association
HE: historical eastern
HTAP: high‐temperature adult plant
IT: infection type
KASP: Kompetitive allele‐specific polymerase chain reaction
LD: linkage disequilibrium
MTA: marker–trait association
QTL: quantitative trait loci
RIL: recombinant inbred line
SE: southeastern elite
SEV: severity
SRWW: soft red winter wheat
SSR: simple sequence repeat.

## 1 INTRODUCTION

Stripe rust, or yellow rust, is a disease caused by the fungal pathogen *Puccinia striiformis* Westend. f. sp. *tritic*. In wheat (*Triticum aestivum* L.), the disease causes chlorotic patches followed by the formation of yellow pustules of uredinia (fungal fruiting bodies) arranged in striped patterns. Uredinia may form on any part of the plant but primarily form on leaves (Chen & Kang, 2017). A recent survey found that stripe rust is a widespread problem in most wheat‐growing regions around the globe, and in the United States, stripe rust outbreaks happen with high frequency and may incur economic losses of 5–10% (Wellings, 2011). Primarily problematic in cooler wheat‐growing regions, *P. striiformis* has historically caused significant crop losses in the western United States (Milus, Kristensen, & Hovmøller, 2008). However, in the year 2000, a large stripe rust outbreak occurred in over 20 states, notably causing severe infections in Arkansas and portions of Texas (Chen et al., 2002). Twenty‐one new races were isolated from this outbreak including the first races to demonstrate virulence against *Yr8* and *Yr9* in the United States (Chen et al., 2002). Further analyses showed that several of the new isolates collected in and since 2000 were better adapted to warmer climates and were therefore more aggressive and destructive in the southern states encompassing the pathogen's new range expansion (Milus et al., 2008; Milus, Seyran, & McNew, 2006). These new isolates tended to have shorter latent periods and higher urediniospore germination at higher temperatures, enabling faster reproduction in warmer environments. The emergence of these new, more aggressive isolates has led to scrutiny of currently deployed sources of disease resistance within the southeastern United States.

One widely utilized stripe rust resistance gene is *Yr17*, which was introgressed from *Ae. ventricosa* (2*n* = 4*x* = 28; D^V^N^V^ genome) into the short arm of wheat chromosome 2A in the development of the French wheat hexaploid line VPM1 (Bariana & McIntosh, 1994). The 2NS:2AS *Ae. ventricosa* introgression harboring *Yr17* also carries the leaf rust (*P. triticina* Erikss.) resistance gene *Lr37*, and the stem rust (*P. graminis* Pers.) resistance gene *Sr38* (Helguera et al., 2003). Bariana and McIntosh (1993) reported that in wheat, *Yr17*, *Lr37*, and *Sr38* occur in tight coupling linkage. No recombination was observed between *Sr38* and *Yr17* in a population of 109 F_2_ plants derived from a cross between VPM1 and Australian cultivar Harrier, and a single recombination was observed between *Lr37* and *Sr38/Yr17*. *Yr17* is classified as an all‐stage resistance (ASR) gene as it confers some amount of disease resistance throughout the lifecycle of the plant. However, *Yr17* resistance is also known to be influenced by several genetic and environmental factors. For example, seedlings containing *Yr17* are more susceptible at lower temperatures and light intensities (Wang & Chen, 2017). In addition, a suppressor of *Yr17* was found to be present in the wheat cultivar Anza, resulting in this line being susceptible to *Yr17*‐avirulent stripe rust isolates despite its possession of the resistance gene (Helguera et al., 2003). Although *Yr17* was originally classified as an ASR gene, it was described as an adult‐plant resistance (APR) gene in ‘Rendezvous’ and other United Kingdom wheat cultivars through 1994, as seedlings often exhibited susceptibility to many isolates to which adult plants were resistant (Milus, Lee, & Brown‐Guedira, 2015). Wan et al. (2016) hypothesized that *Yr17* is closely linked with a high‐temperature adult plant (HTAP) resistance gene also present on the *Ae. ventricosa* 2NS:2AS translocation, and the findings of L. Liu et al. (2018) support this hypothesis. In a genome‐wide association (GWA) panel, they identified both *Yr17* and a separate quantitative trait locus (QTL; *Qyr.wpg‐2A.2*) influencing stripe rust resistance on chromosome 2A, but these two loci were located on separate linkage blocks and demonstrated differential effects on disease resistance across environments. A subsequent study found that the 2NS:2AS introgression conferred APR against *Yr17*‐virulent isolates, further supporting the hypothesis of a HTAP resistance gene in close linkage with *Yr17* (L. Liu et al., 2018). Another resistance gene on 2AS is *YrR61*, which was first described in the SRWW cultivar Pioneer 26R61 (Hao et al., 2011). *YrR61* is an APR gene that is not located on the 2NS:2AS introgression, as Hao et al. (2011) observed that the 2NS:2AS diagnostic simple sequence repeat (SSR) markers *VENTRIUP/LN2_262_* and *Xgwm636_104_* did not amplify in Pioneer 26R61.

Currently, *Yr17* itself offers precarious resistance against stripe rust, as a recent study of 235 worldwide *P. striiformis* isolates found that 88% demonstrated virulence against this gene (Sharma‐Poudyal et al., 2012). *Yr17* has been defeated by most *P. striiformis* isolates in recent years including PSTv‐37, the most predominant isolate in the United States in the last decade (W. Liu et al., 2018). Several recent biparental QTL mapping studies have suggested that additional loci on chromosomes 3BS and 4BL also play an important role in stripe rust resistance in soft winter wheat (Christopher et al., 2013; Liu et al., 2019; Subramanian, Mason, Milus, Moon, & Brown‐Guedira, 2016), though the resistance genes underlying these loci still remain unidentified.

The purpose of the current study was to assess the primary sources of stripe rust resistance in germplasm used for the development of soft red winter wheat (SRWW) elite breeding material in the southeastern United States. To do so, we performed GWA analyses on a panel of historically important soft winter wheat breeding material in environments in Arkansas and the Pacific Northwest, where stripe rust epidemics frequently occur. The findings from the first study were then validated by performing a GWA analysis on a panel of more recent elite breeding material tested in both Arkansas and Georgia.

## 2 MATERIALS AND METHODS

### 2.1 Plant materials and testing environments

The germplasm used in this study consisted of two panels of SRWW. The first was labeled the historical eastern (HE) panel and consisted of 273 lines including a mixture of landraces, older cultivars, and more contemporary breeding lines sourced from public breeding programs across the eastern United States soft winter wheat growing region. The oldest lines in this panel were ‘Goens’, purportedly introduced to Ohio in 1808, and ‘Mediterranean’, believed to have been introduced to Delaware in 1819 (Clark, Martin, & Ball, 1922). The HE panel was tested in a total of eight environments in Washington and Arkansas. These were Central Ferry, WA, in 2013 (CF13), Mount Vernon, WA, in 2013 and 2014 (MV13 and MV14), the Spillman agronomy farm at Washington State University in Pullman, WA, in 2014 (SF14), the Whitlow agronomy farm at Washington State University in 2013 and 2014 (WHIT13 and WHIT14), and the Milo J. Shult Agricultural Research & Extension Center in Fayetteville, AR, in 2013 and 2014 (FAY13 and FAY14). The combination of each testing location and harvest year was considered an environment. Ratings on the HE panel were taken at multiple timepoints throughout the growing season, roughly corresponding to the Zadoks growth stages (GS) (Zadoks, Chang, & Konzak, 1974) of stem elongation (GS 30–39), flowering (GS 65), flowering‐grain fill transition (GS 70–75), and grain fill (GS 75–80). Ratings were averaged across timepoints for each row. Disease development relied on naturally occurring inoculum for most of the HE panel environments. The exceptions were the SF14 and WHIT14 environments, which received artificial inoculations of races PSTv‐14 and PSTv‐37. In these environments, borders and spreader strips were sprayed with inoculum from a backpack sprayer early in the growing season when differentials did not detect the presence of the two races listed above; borders and spreaders subsequently served as the inoculum source through the rest of the growing season. In addition, the FAY13 and FAY14 environments were inoculated as described in Subramanian et al. (2016). Briefly, the FAY13 environment received artificial inoculation with a mixture of *Pst* isolates AR12‐21 and AR13‐01, and the FAY14 environment received artificial inoculation with isolate AR12‐25.

**Core Ideas**

- Southeastern U.S. winter wheat germplasm contains multiple stripe rust resistance loci
- A QTL located on chromosome 4B is important for stripe rust resistance in the Southeast
- The *Yr17* resistance gene on chromosome 2A has been defeated in the southeast for several years
- A *Yr17*‐linked adult plant resistance gene still offers quantitative stripe rust resistance
- The 4B resistance QTL may have unstable effects across environments and sets of germplasm

The second panel was labeled the southeastern elite (SE) panel and consisted of 588 breeding lines included in the cooperative Gulf‐Atlantic Wheat Nursery between the years of 2008 and 2017. This panel was tested in Plains, GA, in 2018 and 2019 (PL18 and PL19) and Fayetteville, AR, in 2018 and 2019 (FAY18 and FAY19). The SE panel primarily contained lines sourced from public breeding programs in Arkansas, Georgia, Florida, Louisiana, North Carolina, South Carolina, Texas, and Virginia beginning in the early 2000s. The HE panel and SE panel shared 36 common lines, and southern accessions from the HE panel are present in the pedigrees of many SE panel accessions. Because of the low percentage of shared lines, analyses were performed on each panel separately, and results were compared post hoc. The SE panel received natural inoculation in the Plains, GA, environments and artificial inoculation with a mixture of the isolates listed in Subramanian et al. (2016) in the Fayetteville, AR, environments.

### 2.2 Phenotypic data collection and analysis

Entries in the HE panel were planted in 1.2‐m‐long single rows, spaced 30 cm apart using a head‐row planter (Wintersteiger, Inc.). Within each environment, up to three replications were planted in a randomized, complete‐block design, the number of replications being based on available space and resources (Table 1). In all the Pacific Northwest environments, the stripe rust‐susceptible check PS 279 was included once per 20 entries. In the 2013 and 2014 Arkansas environments, susceptible check ‘Renegade’ was included once per head‐row tray (i.e. once per 80 entries). In the 2018 and 2019 Arkansas environments, wheat line CG514W was included as a susceptible check once per tray and wheat cultivar Pat was included as a resistant check once per tray. In the 2018 and 2019 Georgia environments, SS‐520 (PI 619052) was included as a susceptible check and USG 3555 (PI 654454) was included as a resistant check, planted once per tray. Stripe rust severity (SEV) was rated on a modified Cobb scale (Peterson, Campbell, & Hannah, 1948) from 0 to 100, representing the percentage of the flag leaf covered by pustules. Infection type (IT) was scored on a scale from 0 to 9 as defined by Line and Qayoum (1992), where 0 corresponds to no visible symptoms, 1–3 correspond to trace sporulation, 4–6 correspond to light to moderate sporulation, and 7–9 correspond to abundant sporulation. Because of the presence of some environments without replications, the genotypic arithmetic means were used in environments with multiple replications.

**TABLE 1:** Summary statistics of stripe rust disease ratings across environments included in the study

Panel^a^	Trait^b^	Environment^c^	Reps	Mean	Median	Min.	Max.	SD
HE	IT	CF13	3	6.25	6.67	2.00	9.00	1.51
		FAY13	3	4.46	4.50	1.00	7.67	1.63
		FAY14	1	3.21	3.00	0	7.50	1.59
		MV13	2	6.07	7.00	2.00	9.00	1.94
		MV14	3	6.23	7.00	1.67	9.00	2.22
		SF13	1	4.29	4.00	2.00	9.00	1.50
		SF14	2	5.92	6.00	1.50	9.00	2.02
		WHIT13	2	6.41	6.00	2.00	9.00	1.24
		WHIT14	2	5.93	6.50	1.00	9.00	2.05
	SEV	CF13	3	50.50	53.33	5.33	90.00	21.23
		FAY13	3	28.99	25.67	0	82.83	22.26
		FAY14	1	14.67	8.50	0	77.50	15.38
		MV13	2	63.91	70.00	5.00	100.00	25.44
		MV14	3	60.66	70.00	5.00	90.00	23.73
		SF14	2	37.74	40.00	2.00	90.00	22.43
		WHIT13	2	64.55	70.00	5.00	100.00	18.67
		WHIT14	2	51.21	55.00	2.00	90.00	22.67
SE	SEV	FAY18^d^	2	28.28	24.25	0	90.75	24.71
		FAY19^d^	2	14.06	5.33	0	97.00	21.17
		PL18^e^	1	1.54	0	0	9.00	2.43
		PL19^e^	2	2.67	2.00	0	9.00	2.50

^a^ HE, Historical eastern panel; SE, southeastern elite panel.

^b^ IT, infection type; SEV, disease severity.

^c^ Environment defined as combination between location (CF, Central Ferry, WA; FAY, Fayetteville, AR; MV, Mount Vernon, WA; PL, Plains, GA; SF, Spitlow agronomy farm, Pullman, WA; WHIT, Whitlow agronomy farm, Pullman, WA) and two‐digit year.

^d^ Data collected on 1–100 scale; converted to within‐environment percentiles prior to further analysis.

^e^ Data collected on 0–9 scale; converted to within‐environment percentiles prior to further analysis.

Entries in the SE panel were planted in single rows without replication in the PL18 environment and with two replications in the PL19, FAY18, and FAY19 environments (Table 1). Stripe rust severity was rated on a modified Cobb scale in the Arkansas environments, and on a 0–9 scale in the Georgia environments. All scores were converted to percentiles within each environment where the SE panel was grown to standardize ratings. As with the HE panel, the within‐environment arithmetic means were used for calculation of the across‐environment adjusted means in the SE panel because of the presence of the unreplicated PL18 environment.

The following mixed linear model was fit to the phenotypic data for each panel separately:

(1) $$y\;=\;\mu +G_{i}+E_{j}+\varepsilon_{\mathrm{ij}}$$

where phenotypic response (*y*) is a function of the overall mean (μ), the *i*th genotype (*G_i_*) in the *j*th environment (*E_j_*), and the residual error (ε*_ij_*). Genotype was treated as a fixed effect and environment was treated as a random effect. Equation 1 was used to calculate genotypic best linear unbiased estimators (BLUEs) across environments. In order to calculate entry mean heritability, the model described in Equation 1 above was used but treating both genotype and environment as random effects. Heritability was then estimated for each trait as follows:

(2) $$H^{2}=\frac{\sigma_{g}^{2}}{\left(\sigma_{\varepsilon }^{2}/E\right)+\sigma_{g}^{2}}$$

Where entry mean heritability (*H* ^2^) is a function of genotypic variance ($\sigma_{g}^{2}$), residual variance ($\sigma_{\varepsilon }^{2}$), and the number of environments (*E*).

### 2.3 Genotyping

Seeds of each genotype were germinated, and genomic DNA was isolated from seedling leaf tissue on an LGC Biosearch Technologies (Teddington, Middlesex, UK) Oktopure robotic extraction platform, using sbeadex magnetic microparticle reagent kits. Genotyping‐by‐sequencing was performed on an Illumina (San Diego, CA) HiSeq 2500 sequencer following a *PstI‐MspI* double restriction digest of genomic DNA (Poland, Brown, Sorrells, & Jannink, 2012). Illumina reads were aligned to the International Wheat Genome Sequencing Consortium ‘Chinese Spring’ v1.0 reference sequence (International Wheat Genome Sequencing Consortium et al., 2018) using the Burrows–Wheeler Aligner v0.7.17 (Li & Durbin, 2009). Single nucleotide polymorphism calling was performed for both panels jointly using TASSEL‐GBS in TASSEL 5.2.50 (Bradbury et al., 2007; Glaubitz et al., 2014). The raw SNP call data was then filtered to remove SNPs with missing data frequency >50%, heterozygous call frequency >10%, or minor allele frequency <5%. Single nucleotide polymorphisms located on unassembled contigs were discarded. After the initial filtering, missing data in the genotypic dataset was imputed and phased using Beagle 5.0 (Browning & Browning, 2007; Browning, Zhou, & Browning, 2018). To assess imputation accuracy, 10,000 randomly selected nonmissing genotype calls were masked and imputed along with all missing data in the dataset. Following imputation, imputed genotype calls were compared against their corresponding known calls, and the proportion of correctly imputed calls was calculated. The dataset was filtered a second time following imputation to remove SNPs with minor allele frequencies <5%. The imputed genotypic dataset was then filtered in PLINK 1.9 (Chang et al., 2015) to remove all but one SNP from groups of SNPs in perfect linkage disequilibrium (LD; *r* ^2^ = 1) using a 250‐SNP sliding window, advancing by 10 SNPs with each step. After filtering and LD thinning, 61,268 SNPs remained for further analysis.

Previously collected data from a targeted Kompetitive allele‐specific polymerase chain reaction (KASP) assay was compiled to determine the presence or absence of the 2NS:2AS translocation carrying the *Yr17/Lr37/Sr38* locus. Of the 588 total lines included in the SE panel, 310 (52.7%) were genotyped with the KASP 2NS:2AS assay. In the HE panel, 252 of the total 273 lines (92.3%) were genotyped with the 2NS:2AS assay. In addition, we compiled KASP assay data to interrogate the presence or absence of the *Sr2* stem rust resistance gene and linked *Yr30* stripe rust resistance gene on chromosome 3BS in 244 of the 273 lines (89.3%) included in the HE panel. Primer sequences for both assays are listed in Supplemental Table S1. Pairwise LD estimates (*r* ^2^) were calculated between the KASP assays and each GBS SNP located on the same chromosome. Finally, we also used a total of six KASP assays developed by Liu et al. (2019) to characterize significant marker–trait associations (MTAs) on chromosomes 3BS and 4BL. These assays were based on the Illumina iSelect 90K wheat SNP array (Wang et al., 2014) SNPs IWB1836, IWB20761, and IWA7230 (3BS) and IWB33055, IWB13720, and IWB433 (4BL). Markers of Liu et al. (2019) were tested in a total of 685 lines: 233 from the HE panel, 415 from the SE panel, and 37 shared between both panels (Supplemental Table S2). The KASP data contained low levels of missing data (2.94%), which was imputed categorically using the missForest package (Stekhoven & Bühlmann, 2012) in the R statistical computing environment (R Core Team, 2015). Lines containing heterozygous calls for any of the six KASP SNPs were removed prior to analysis.

### 2.4 Population structure

In order to determine whether any cryptic population structure was present within each of the panels, we first created more stringently filtered genotypic datasets. For both the HE and SE panels, we filtered the genotypic datasets to remove all SNPs with >20% missing data, >10% heterozygous calls, or <5% minor allele frequency. We then used Plink 1.9 –indep‐pairwise option to create subsets of SNPs such that no pair of SNPs on a given chromosome exhibited a pairwise LD estimate (*r* ^2^) over .15. Following these filtering steps, the HE panel dataset contained 4,710 SNPs, while the SE panel dataset contained 7,489 SNPs. We then used the software ADMIXTURE 1.3 (Alexander, Novembre, & Lange, 2009) to determine whether population structure was present in each panel. To determine an ideal number of subpopulations (*k*) within each panel, we measured the prediction error of subpopulation inclusion probabilities (i.e. *Q*‐matrix coefficients) using fivefold cross‐validation as recommended by Alexander and Lange (2011), incrementally increasing *k* from 1 to 10. Following identification of an optimal number of clusters corresponding to the minimum prediction error for each panel, we clustered lines into subpopulations by performing partitioning around medoids using the ideal *k* value in the R package ‘cluster’ (Maechler, Rousseeuw, Struyf, Hubert, & Hornik, 2019) and the ADMIXTURE‐generated Q‐matrix. Principal component analysis was performed using smartpca (Patterson, Price, & Reich, 2006) on a SNP matrix filtered using the same parameters listed above. However, in this case, the two panels were processed and filtered jointly.

### 2.5 Genome‐wide association analyses

Preliminary single‐locus mixed linear model GWA analyses were conducted on both panels using Genome‐Wide Complex Trait Analysis (GCTA) software (Yang, Lee, Goddard, & Visscher, 2011). However, the 2NS:2AS translocation produced severe inflation of SNP *p* values. This inflation could potentially mask other significant loci on chromosome 2A. Therefore, a multi‐locus mixed linear model (Segura et al., 2012) was used to fit up to eight significant SNPs as covariates, eliminating the background *p* value inflation caused by the 2NS:2AS translocation. For each panel, the [samples *×* SNPs] matrix was recoded into a numeric minor‐allele dosage format (i.e. 0 corresponding to a homozygous major allele call, 1 corresponding to a heterozygous call, and 2 corresponding to a homozygous minor allele call). Based upon the population structure analysis described above, a four‐column *Q* matrix of ADMIXTURE‐generated subpopulation inclusion probabilities was supplied to model population structure in the HE panel in addition to the genomic relationship matrix, while only the genomic relationship matrix was used to model population structure in the SE panel.

### 2.6 Estimation of variance explained by markers

For each environment–trait combination yielding significant results in the GWA analysis step, a linear model was fit including the *Q* matrix used to model population structure (if any) as well as all significant SNPs. The R package ‘relaimpo’ (Groemping, 2006) was used to estimate the proportion of phenotypic variance explained by each significant SNP. Genome‐based restricted maximum likelihood modeling was then performed in GCTA (Yang et al., 2010, 2011) to estimate the proportion of phenotypic variance explained by all SNPs jointly.

### 2.7 Haplotype analysis

For the major resistance QTL occurring on chromosomes 2AS, 3BS, and 4BL, haplotype block regions surrounding significant MTAs for the trait SEV were delimited using the positions of the GWA‐identified significant SNPs reported from the multi‐locus mixed model across both panels and all environments. A discriminant analysis of principal components (Jombart, Devillard, & Balloux, 2010) was performed on SNPs within each QTL region using R package ‘adegenet’ (Jombart, 2008; Jombart & Ahmed, 2011) to cluster genotypes into haplotypes, allowing up to a maximum of 15 clusters. The number of clusters for each haplotype was determined with the nbclust() function in the R package ‘factoextra’ (Kassambara & Mundt, 2020), using hierarchical clustering. Analysis of variance models were run for each panel, fitting terms for the three major QTL regions, the three pairwise epistatic interactions between them, and the single three‐way epistatic interaction. Tukey's honestly significant difference test was performed for each major QTL region in each panel; haplotypes of each QTL were assigned to groups ranging from susceptible to resistant based upon their marginal means. Two haplotypes were assigned to the same group, for example, two resistance haplotypes for a single QTL if their phenotypic means were not found to be significantly different.

## 3 RESULTS

### 3.1 Imputation accuracy and population structure

**FIGURE 1:**
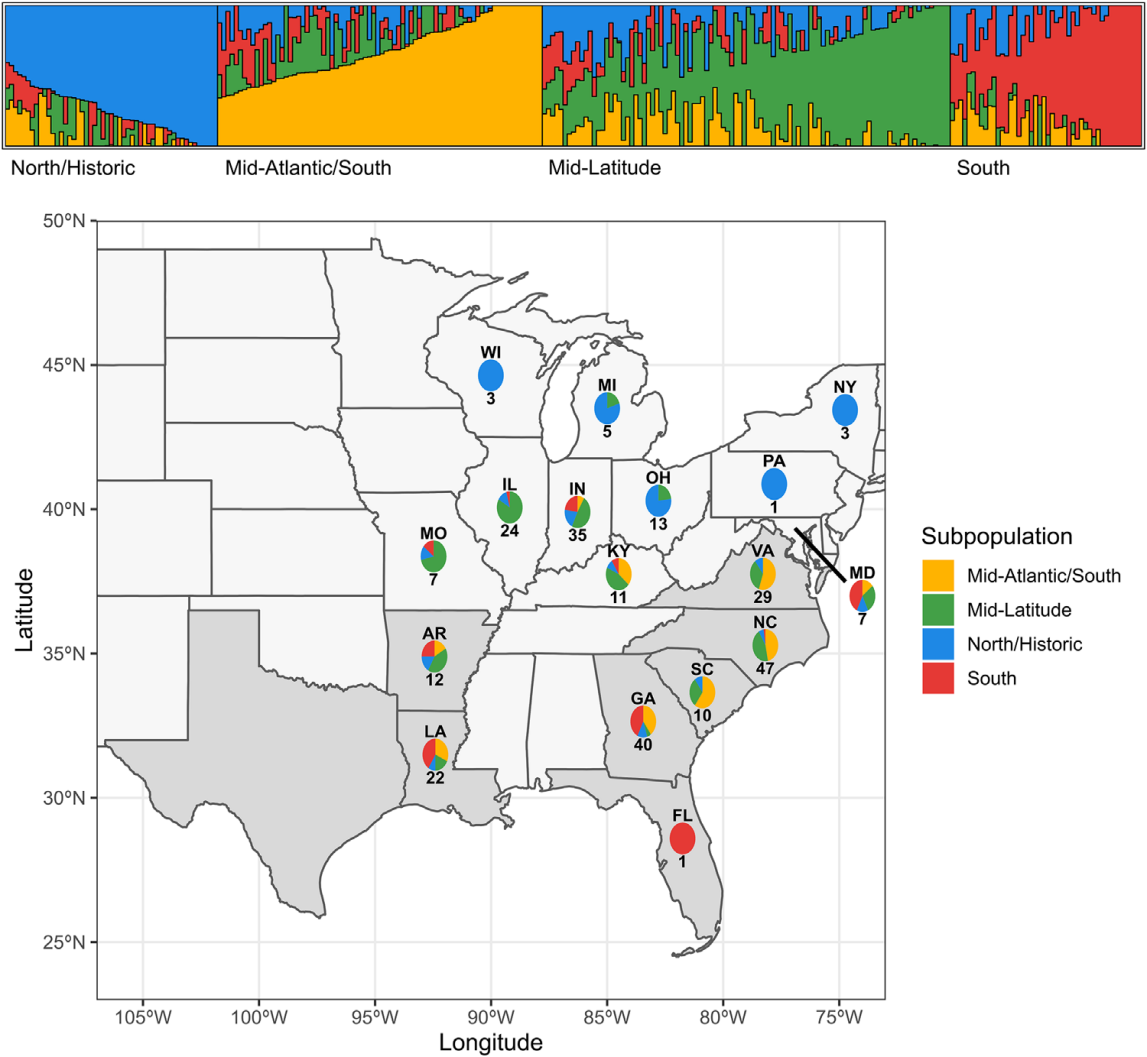
Source states and subpopulations for the historical eastern panel (HEP) and southeastern elite panel (SEP). Top: Admixture subpopulation inclusion probabilities for *k* = 4 subpopulations in the HEP; no significant population structure was detected in the SEP. Bottom: Proportion of HEP lines assigned to each subpopulation within each state of origin. The total number of lines sourced from each state is listed below each pie chart. States from which SEP lines were also sourced are shaded darker gray

We found that Beagle produced a consistently high accuracy of imputation, largely independent of a given SNP's proportion of missing data or minor allele frequency (Supplemental Figure S1). In general, Beagle imputed missing data correctly >95% of the time, the only exception being a mean accuracy of 93.4% for SNPs with 40–50% missing data and minor allele frequency between .2 and .3. Results of the ADMIXTURE cross‐validation procedure indicated a minimum error with *k* = 4 for the HE panel. The SE panel did not exhibit any distinct minimum in cross‐validation error up to a *k* value of 10, and hence we determined that there was no significant population structure present in this panel (Supplemental Figure S2). Bar plots of ADMIXTURE‐derived subpopulation inclusion probabilities for each line in the HE panel are shown in the top panel of Figure 1. The partitioning‐around‐medoids algorithm identified four clusters in the HE panel that roughly corresponded to geographic regions of origin: one group representing the southern region (primarily Louisiana, Georgia, and Arkansas), one for the Mid‐Atlantic–southern region (primarily Louisiana, Georgia, South Carolina, North Carolina, Virginia, and Kentucky), one consisting primarily of lines from mid‐latitude states including the Midwest, and one consisting of primarily northern and historical lines (bottom panel of Figure 1). The principal component analysis of the joint, LD‐thinned SNP dataset confirmed that the SE panel is more closely related to the southern accessions present in the HE panel (Supplemental Figure S3).

### 3.2 Phenotypic response across environments

Disease pressure was generally consistent across most environments within each panel (Table 1). For the HE panel, disease severity was low in the FAY14 environment, with a mean of 14.67 on a 0–100 rating scale, but ranged from 28.99 to 64.55 in all other environments. Disease ratings were generally lower in the SE panel, with mean severities of 28.28 and 14.06 in the FAY18 and FAY19 environments, respectively (0–100 rating scale), and mean severities of 1.54 and 2.67 in the PL18 and PL19 environments, respectively (0–9 rating scale). The across‐environment entry mean heritabilities for SEV and IT ratings in the HE panel were .93 and .94, respectively (Supplemental Table S3), while the across‐environment entry mean heritability for SEV ratings in the SE panel was .82. Because of the low number of lines shared between both panels (36), disease IT and SEV ratings were not directly comparable between panels, and differences in environmental disease pressure and genetic disease resistance between them remained confounded. The conversion of SE panel SEV scores to within‐environment percentiles also prevented direct comparisons between the two panels.

### 3.3 Genome‐wide association studies

**FIGURE 2:**
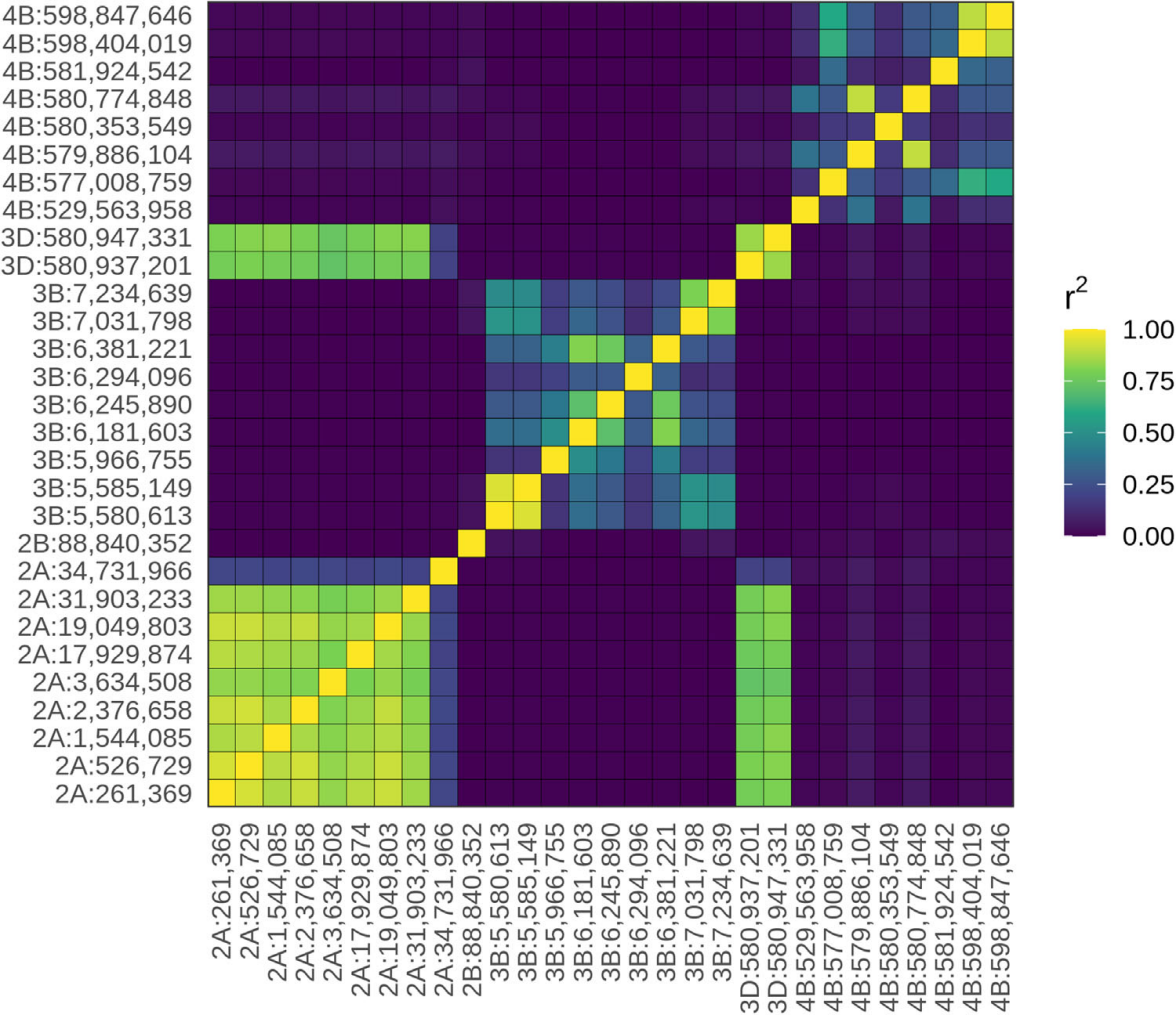
Pairwise linkage disequilibrium estimates (*r* ^2^) between all significant marker–trait associations identified by the multi‐locus mixed linear model for all traits and environments tested among both panels

In the HE panel, the multi‐locus mixed model identified a total of 42 significant MTAs for IT and SEV within individual environments and across environments (Table 2). In contrast, 13 significant MTAs were identified in the SE panel (Table 3). Marker–trait associations across and within environments were mostly located within three major QTL regions: chromosome 2AS, ranging from the distal end of the short arm to ∼32 Mb; a region on 3BS from ∼5.6 to 7.2 Mb; and a region on 4BL from ∼529.5 to 599 Mb. The multi‐locus mixed model selected multiple SNPs within these regions for different environments, though the pairwise LD between all significant MTAs clearly delineated these three major regions (Figure 2). Pairwise LD was generally higher within the 2AS QTL than the 3BS and 4BL QTL.

**TABLE 2:** Significant marker–trait associations for the traits infection type (IT) and severity (SEV) across and within testing environments of the historic eastern panel

Environment^a^	Trait^b^	Chromosome	Position	Distance^c^	Alleles^d^	MAF^e^	*P* value	PVE^f^
			bp	cM				
ACROSS	IT	2A	261,369	0	A, G	.12	4.68 × 10^−16^	.21
ACROSS	IT	3B	7,234,639	5.85	A, G	.42	1.29 × 10^−8^	.09
ACROSS	IT	4B	580,774,848	50.16	T, C	.30	9.89 × 10^−12^	.10
ACROSS	SEV	2A	261,369	0	A, G	.12	5.20 × 10^−22^	.27
ACROSS	SEV	3B	5,580,613	3.79	G, T	.39	9.48 × 10^−7^	.07
ACROSS	SEV	4B	579,886,104	50.12	C, T	.29	1.39 × 10^−14^	.11
ACROSS	SEV	4B	580,353,549	50.14	A, G	.39	1.93 × 10^−6^	.04
CF13	IT	2A	261,369	0	A, G	.12	9.00 × 10^−12^	.15
CF13	IT	4B	598,404,019	51.15	G, T	.32	1.52 × 10^−8^	.13
CF13	SEV	2A	261,369	0	A, G	.12	1.61 × 10^−11^	.15
CF13	SEV	4B	598,404,019	51.15	G, T	.32	4.06 × 10^−8^	.12
FAY13	IT	3D	580,937,201^g^	120.54	C, T	.15	2.73 × 10^−12^	.20
FAY13	IT	4B	598,404,019	51.15	G, T	.32	1.71 × 10^−10^	.15
FAY13	SEV	2B	88,840,352	56.49	T, C	.21	2.49 × 10^−6^	.03
FAY13	SEV	3D	580,937,201^g^	12.54	C, T	.15	1.11 × 10^−9^	.16
FAY13	SEV	4B	598,404,019	51.15	G, T	.32	6.29 × 10^−9^	.14
FAY14	IT	2A	34,731,966	23.48	C, T	.10	2.07 × 10^−6^	.07
FAY14	IT	3D	580,947,331^g^	120.54	A, G	.16	1.78 × 10^−7^	.12
FAY14	IT	4B	577,008,759	49.97	G, C	.27	3.38 × 10^−10^	.12
FAY14	SEV	4B	529,563,958	46.32	C, T	.22	8.44 × 10^−8^	.11
MV13	IT	3B	6,381,221	4.80	A, G	.26	6.07 × 10^−10^	.13
MV13	IT	3D	580,937,201^g^	120.54	C, T	.15	2.02 × 10^−10^	.13
MV13	IT	4B	598,404,019	51.15	G, T	.33	1.03 × 10^−9^	.14
MV13	SEV	2A	31,903,233	21.42	A, C	.13	1.19 × 10^−13^	.18
MV13	SEV	3B	6,181,603	4.55	G, C	.30	4.10 × 10^−8^	.10
MV13	SEV	4B	598,404,019	51.15	G, T	.33	2.93 × 10^−10^	.13
MV14	IT	2A	261,369	0	A, G	.12	6.06 × 10^−20^	.21
MV14	IT	3B	7,031,798	5.60	A, C	.46	1.15 × 10^−6^	.05
MV14	IT	4B	598,404,019	51.15	G, T	.32	1.56 × 10^−14^	.16
MV14	SEV	2A	261,369	0	A, G	.12	1.45 × 10^−16^	.23
MV14	SEV	4B	598,404,019	51.15	G, T	.32	2.31 × 10^−9^	.13
SF14	IT	2A	17,929,874	9.84	T, A	.11	3.83 × 10^−11^	.16
SF14	IT	3B	6,294,096	4.69	A, C	.10	4.50 × 10^−8^	.07
SF14	IT	4B	580,774,848	50.16	T, C	.30	6.83 × 10^−7^	.05
SF14	SEV	3B	5,580,613	3.79	G, T	.39	1.35 × 10^−6^	.08
SF14	SEV	3D	580,937,201^g^	120.54	C, T	.15	2.34 × 10^−13^	.20
WHIT13	SEV	2A	526,729	0	A, T	.12	1.56 × 10^−20^	.28
WHIT13	SEV	4B	581,924,542	50.22	C, T	.13	6.16 × 10^−7^	.06
WHIT14	IT	2A	1,544,085	0	C, A	.12	2.47 × 10^−15^	.23
WHIT14	IT	3B	5,585,149	3.80	A, G	.39	5.02 × 10^−7^	.08
WHIT14	SEV	2A	1,544,085	0	C, A	.12	1.90 × 10^−18^	.26
WHIT14	SEV	3B	5,585,149	3.80	A, G	.39	1.06 × 10^−7^	.08

^a^ ACROSS, genotypic best linear unbiased estimators calculated across environments; CF13, Central Ferry, WA, 2013; FAY13, Fayetteville, AR, 2013; FAY14, Fayetteville, AR, 2014; MV13, Mount Vernon, WA, 2013; MV14, Mount Vernon, WA, 2014; SF14, Spitlow agronomy farm, Pullman, WA, 2014; WHIT13, Whitlow agronomy farm, Pullman, WA, 2013; WHIT14, Whitlow agronomy farm, Pullman, WA, 2014.

^b^ IT, disease infection type; SEV, disease severity.

^c^ Genetic distance interpolated from Opata × Synthetic W7984 population described in Gutierrez‐Gonzalez et al. (2019).

^d^ Major allele listed first; advantageous (i.e. resistance) allele underlined.

^e^ PVE, proportion of phenotypic variance explained.

^f^ MAF, minor allele frequency.

^g^ SNP exhibiting high LD with other SNPs located in the region of the Ae. ventricosa 2NS:2AS alien introgression; probable misalignment.

**TABLE 3:** Significant marker–trait associations for disease severity across and within testing environments of the southeastern elite panel

Environment^a^	Chromosome	Position	Distance^b^	Alleles^c^	MAF^d^	*P* value	PVE^e^
		bp	cM				
ACROSS	2A	3,634,508	0	C, G^f^	.50	2.16 × 10^−^13	.10
ACROSS	3B	6,245,890	4.63	T, C	.16	4.61 × 10^−^10	.06
ACROSS	4B	598,404,019	51.15	G, T	.39	1.95 × 10^−^29	.28
FAY18	2A	19,049,803	10.86	A, G ^f^	.50	6.63 × 10^−^13	.10
FAY18	3B	5,966,755	4.28	T, C	.28	3.29 × 10^−^9	.04
FAY18	4B	598,404,019	51.15	G, T	.4	2.75 × 10^−^25	.28
FAY19	2A	3,634,508	0	C, G^f^	.50	1.72 × 10^−^17	.14
FAY19	3B	6,181,603	4.55	G, C	.19	7.45 × 10^−^7	.04
FAY19	4B	598,847,646	51.18	C, G	.39	2.70 × 10^−^21	.17
PL18	4B	598,847,646	51.18	C, G	.38	3.12 × 10^−^9	.13
PL19	2A	2,376,658	0	A, G	.48	1.20 × 10^−^11	.06
PL19	3B	6,245,890	4.63	T, C	.16	1.87 × 10^−^07	.04
PL19	4B	598,404,019	51.15	G, T	.39	4.63 × 10^−^20	.19

^a^ ACROSS, genotypic best linear unbiased estimators calculated across environments; FAY18, Fayetteville, AR, 2018; FAY19, Fayetteville, AR, 2019; PL18, Plains, GA, 2018; PL19, Plains, GA, 2019.

^b^ Genetic distance interpolated from Opata × Synthetic W7984 population described in Gutierrez‐Gonzalez et al. (2019).

^c^ Major allele listed first; advantageous (i.e. resistance) allele underlined.

^d^ MAF, minor allele frequency.

^e^ PVE, proportion of phenotypic variation explained.

^f^ Designation of major vs. minor allele is arbitrary; designation of advantageous allele still applies.

Several MTAs were identified in the HE and SE panels in addition to the three major QTL regions mentioned above. One MTA, located at 2A:34,731,966 bp, affected the trait SEV in the FAY14 environment of the HE panel. This MTA was in moderate LD with the other MTAs in the 2AS major QTL region. One MTA located at 2B:88,840,352 bp affected the trait SEV in the FAY13 environment of the HE panel. Finally, two MTAs were identified on chromosome 3D in the HE panel. One, located at 580,937,201 bp, affected the traits IT and SEV in the FAY13 environment, as well as the trait IT in the MV13 environment and the trait SEV in the SF14 environment of the HE panel. The other, located at 580,947,331 bp, affected the trait IT in the FAY14 environment of the HE panel. However, both of these MTAs exhibited high LD with MTAs in the 2AS QTL, making it likely that they are located on misaligned reads belonging to the 2NS:2AS translocation.

### 3.4 Proportion of variance explained by SNPs

In the HE panel, significant MTAs falling within the 2NS:2AS *Ae. ventricosa* translocation generally explained the largest proportion of phenotypic variance, with values of .27 for SEV and .21 for IT across environments. The 3BS and 4BL loci both generally explained less variance, with the 3BS locus explaining a maximum proportion of variance of .13 for the trait IT measured in the MV13 environment. The 3BS locus explained .09 and .07 of phenotypic variation across environments for the traits IT and SEV, respectively. The 4BL locus explained a maximum of .16 of phenotypic variance for the trait IT in the MV14 environment. Across environments, it explained .10 and .11 of phenotypic variance for the traits IT and SEV, respectively.

The proportion of phenotypic variance explained by all SNPs jointly ranged from .22 for the trait IT measured in the SF13 environment to .64 for the trait IT measured in the SF14 environment and for the traits IT and SEV measured in the WHIT13 environment (Supplemental Table S4). The proportion of phenotypic variance explained by all SNPs jointly for the across‐environment BLUEs was .62 for the trait IT and .57 for the trait SEV. In most environments, the MTAs on 2AS, 3BS, and 4BL jointly accounted for greater than half of the variance accounted for by all SNPs. No significant MTAs were identified for IT measured in the SF13 or WHIT13 environments, and hence all phenotypic variance in these environments was attributed to polygenic background effect. The 2AS QTL was categorized as significant in the GWA analysis for all trait–environment combinations except SEV in FAY14. It therefore did not contribute any estimable portion of variance above the polygenic background for this trait–environment combination. The two MTAs assumed to be misaligned to chromosome 3D were considered as belonging to the 2AS QTL for these calculations. Likewise, the 3BS QTL did not exhibit any significant effects for IT measured in the CF13, FAY13, or FAY14 environments, nor for SEV measured in the CF13, FAY13, FAY14, or MV14 environments. Finally, the 4BL QTL did not exhibit any significant effects for IT in the SF13 or WHIT14 environments, nor for SEV in the SF14 or WHIT14 environments.

**FIGURE 3:**
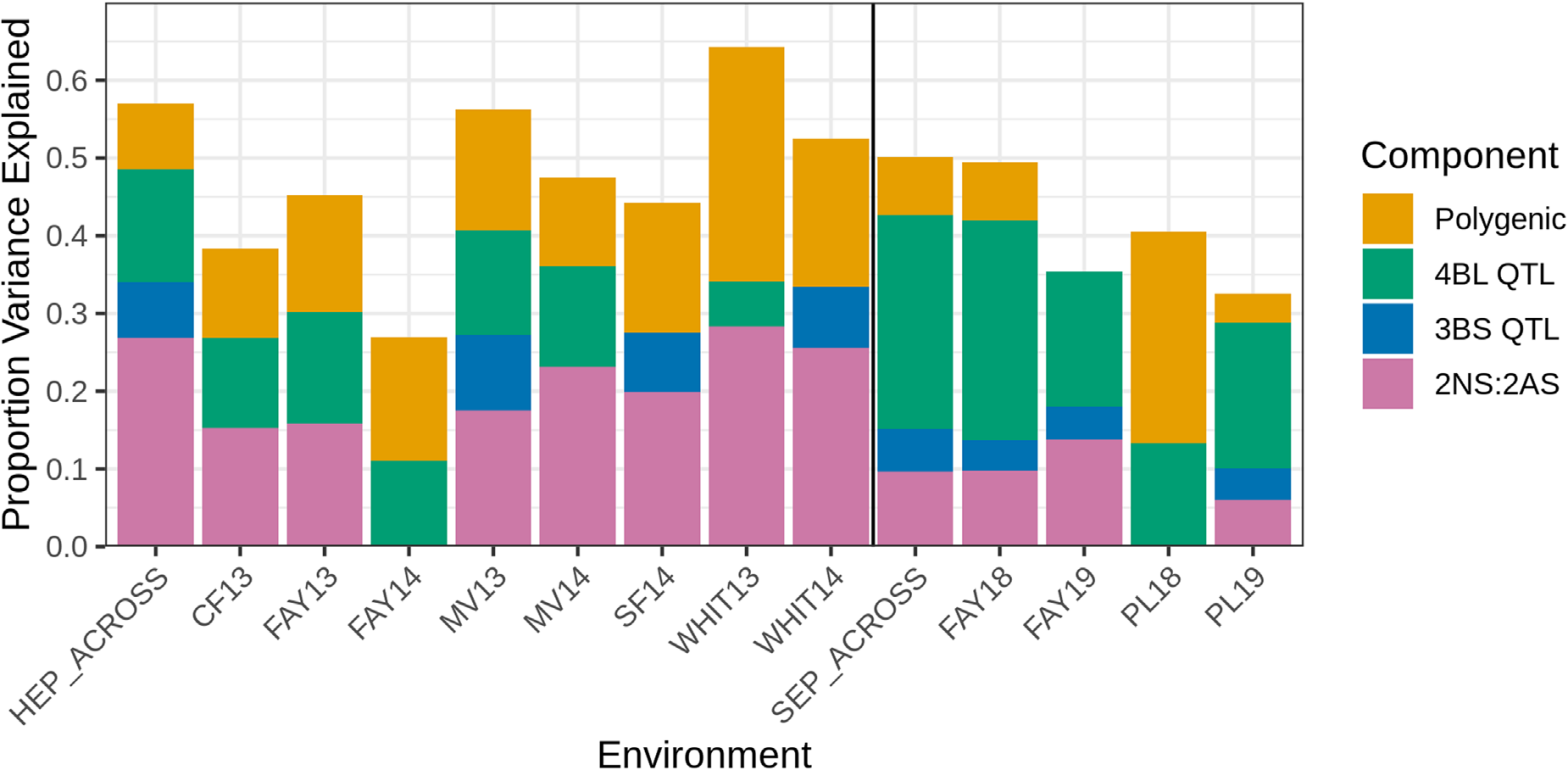
Proportion of disease severity explained by the three major resistance quantitative trait loci (QTL) (2NS:2AS, 3BS, and 4BL) and polygenic background effect. Bars to the left of the vertical dividing line represent across and within‐environment phenotypic variances of the historic eastern panel (HEP); bars to the right of the vertical dividing line represent across and within‐environment phenotypic variances of the southeastern elite panel (SEP). Environments are represented as the combination of location and harvest year. ACROSS, genotypic best linear unbiased estimators calculated across environments; CF, Central Ferry, WA; FAY, Fayetteville, AR; MV, Mount Vernon, WA; PL, Plains, GA; SF, Spitlow agronomy farm, Pullman, WA; WHIT, Whitlow agronomy farm, Pullman, WA

In the SE panel, the SEV variance explained by all SNPs ranged from .31 for the FAY19 environment to .5 for the across‐environment BLUEs (Supplemental Table S4). Neither the 2NS:2AS translocation nor the 3BS QTL exhibited any significant effects on SEV in the PL18 environment, which had low disease pressure. The polygenic background effect did not contribute any additional observable variance in SEV beyond that of the three major QTL in the FAY19 environment. The 2NS:2AS translocation explained a larger proportion of phenotypic variance for SEV in the HE panel when compared with the SE panel, where the 4BL QTL generally explained the most variance (Figure 3).

### 3.5 Haplotypes and allelic combinations

Significant differences in SEV ratings (*p* value < .05) were observed for all three QTL main effects in both panels (Supplemental Tables S5 and S6). No significant epistatic interactions between the major QTLs were found in the HE panel, whereas both the 2AS:3BS and 3BS:4BL interactions were classified as significant in the SE panel (*p* values of .026 and .023, respectively). Distinct haplotypes were detected in both panels for all three major QTL regions (Table 4). For the 2AS QTL, two haplotypes were observed, corresponding to the presence or absence of the 2NS:2AS translocation (Supplemental Figure S4). In the HE panel, the 2NS:2AS translocation occurred with a frequency of 12%, while in the SE panel it occurred with a frequency of 48%. The 3BS QTL displayed three prominent haplotypes (Supplemental Figure S5). The effects of these haplotypes were stable across both panels, with 3BS_1 acting as a susceptibility allele and both 3BS_2 and 3BS_3 acting as resistance alleles with undifferentiated means. The combined frequencies of the 3BS_2 and 3BS_3 resistance alleles were 72% in the HE panel and 83% in the SE panel.

**TABLE 4:** Haplotypes of the three major quantitative trait loci regions located on chromosomes 2AS, 3BS, and 4BL

Panel	Locus	Haplotype	*N*	Frequency	Mean	SE	HSD^a^	Groups^b^
Historic eastern	2AS	2AS_1	241	.88	50.0	15.5	A	S (WT)
		2AS_2	32	.12	21.0	12.3	B	R (2NS:2AS)
	3BS	3BS_1	76	.28	54.6	15.3	A	S
		3BS_2	52	.19	47.2	16.6	B	R
		3BS_3	145	.53	42.1	18.0	B	R
	4BL	4BL_1	58	.21	55.8	16.8	A	S
		4BL_2	154	.56	44.3	17.2	B	R
		4BL_3	24	.09	45.5	17.0	B	R
		4BL_4	37	.14	42.0	17.7	B	R
Southeastern elite	2AS	2AS_1	305	.52	64.4	17.8	A	S (WT)
		2AS_2	285	.48	56.4	14.0	B	R (2NS:2AS)
	3BS	3BS_1	100	.17	70.1	16.3	A	S
		3BS_2	110	.19	56.0	16.0	B	R
		3BS_3	380	.64	59.3	15.9	B	R
	4BL	4BL_1	128	.22	68.4	15.6	A	S
		4BL_2	299	.51	55.1	16.4	C	R
		4BL_3	88	.15	67.4	11.6	A	S
		4BL_4	75	.13	60.8	15.5	B	I

^a^ Results of Tukey's honestly significant difference (HSD) test; entries with different letters have significantly different means.

^b^ WT, wild‐type (susceptible) allele; 2NS:2AS *Ae. ventricosa* 2NS:2AS translocation (resistance) allele; S, susceptibility allele; R, resistance allele; I, intermediate resistance–susceptibility allele.

Finally, the 4BL QTL contained the greatest haplotypic diversity of the three QTL with four haplotypes (Supplemental Figure S6), with some haplotypes displaying variable effects across the two panels. In the HE panel, all but one haplotype (4BL_1) were categorized as resistance haplotypes that occurred at a combined frequency of 79%. The phenotypic effects of the 4BL QTL were more complex in the SE panel, where only the 4BL_2 haplotype was categorized as a resistance allele occurring at a frequency of 51%. Haplotype 4BL_4 was classified as an intermediate resistance–susceptibility allele and occurred at a frequency of 13%. While the 4BL_3 haplotype functioned as a resistance allele in the HE panel, it functioned as a susceptibility allele in the SE panel occurring at a frequency of 15%. Therefore, the only two 4BL haplotypes exhibiting stable effects across both panels were the 4BL_1 susceptibility allele and the 4BL_2 resistance allele.

**FIGURE 4:**
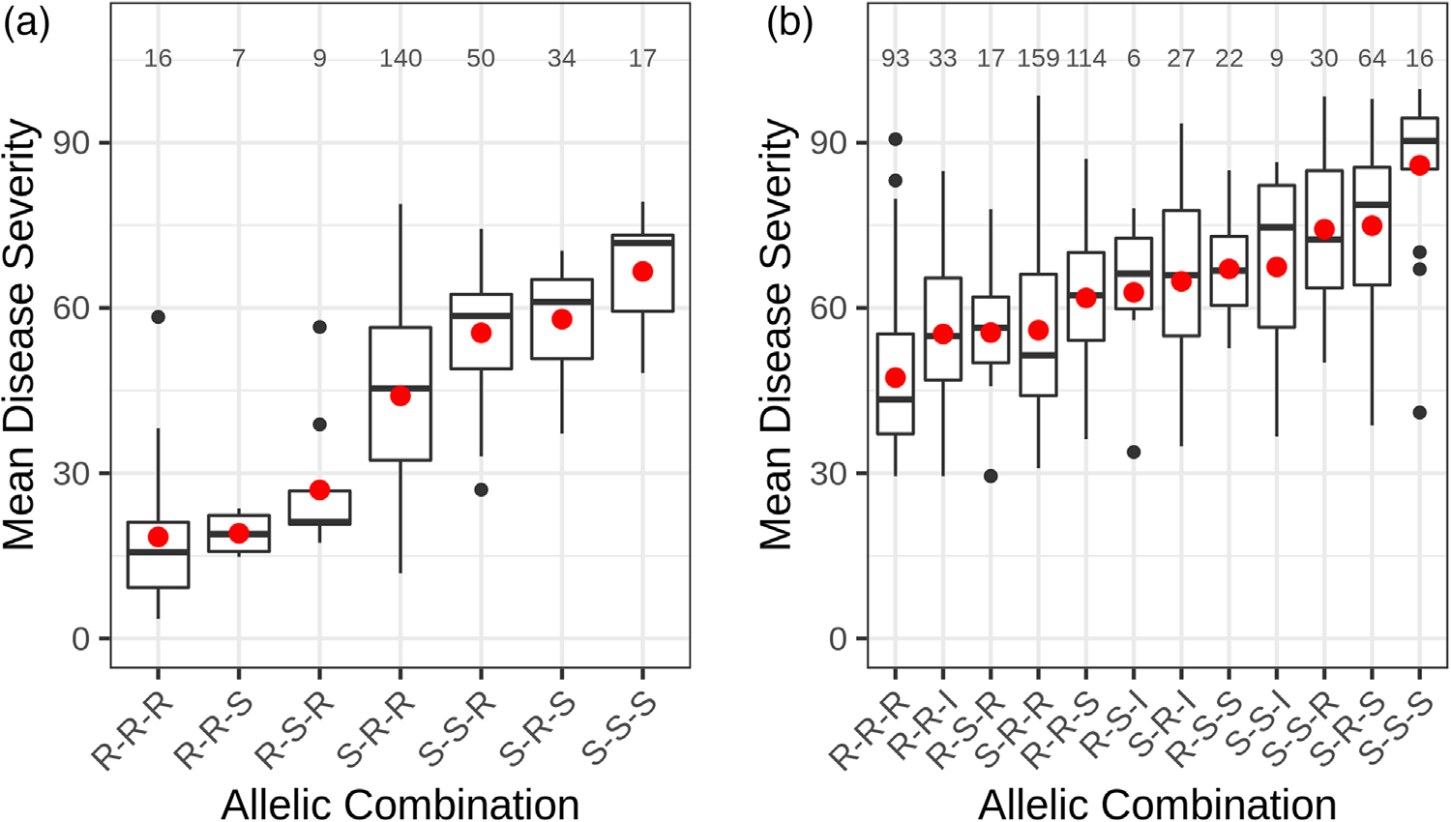
Mean disease severity scores for lines containing different combinations of alleles from the three major resistance quantitative trait loci (QTL) in (A) the historic eastern panel and (B) the southeastern elite panel. Disease severity is not directly comparable between panels. The number of lines containing each combination of alleles is displayed above the boxes. Central red dots indicate the mean for each allelic combination. The combination of alleles is presented in the format 2AS‐3BS‐4BL. S, susceptibility allele; R, resistance allele; I, intermediate susceptibility–resistance allele

The HE panel included a total of seven different combinations of alleles at the three major QTL (Supplemental Table S7), displaying a range of phenotypic means for SEV (Figure 4b). Lines containing the 2NS:2AS translocation consistently demonstrated lower SEV scores than lines containing the wild‐type 2AS allele, while the effects of the other two QTL were less pronounced. Sixteen lines (5.9%) contained resistance alleles for all three QTL and displayed the lowest mean across‐environment SEV score of 18.4. Seventeen lines (6.2%) contained the susceptibility alleles at all three loci, displaying the highest mean across‐environment SEV score of 64.7. The most common allelic combination was the wild‐type 2AS locus paired with resistance alleles at the 3BS and 4BL loci, which occurred in 140 (51.3%) of HE panel lines.

The SE panel included a total of 12 allelic combinations for the three major QTLs (Supplemental Table S8; Figure 4b). Ninety‐three lines (15.8%) contained resistance alleles at all three major QTL, and these lines displayed the lowest mean across‐environment SEV score of 47.3 (percentile score). In contrast, 16 lines (2.7%) contained all three susceptibility alleles and displayed the highest mean across‐environment SEV score of 85.9. The effects of the 2NS:2AS translocation on disease severity were less pronounced in the SE panel than in the HE panel. In addition, the 3BS and 4BL QTL and their interaction explained a larger proportion of variance for SEV. Once again, the most common combination of alleles was the wild‐type 2AS allele in combination with resistance alleles at the 3BS and 4BL loci occurring in 159 (26.9%) of lines. Lines with this grouping of alleles demonstrated lower mean SEV scores than some lines containing the 2NS:2AS translocation. Interaction plots for the 2AS and 3BS loci (Supplemental Figure S7) and the 3BS and 4BL loci (Supplemental Figure S8) in the SE panel demonstrate significant epistatic interaction, with crossover interaction apparent between the 3BS and 4BL loci.

**FIGURE 5:**
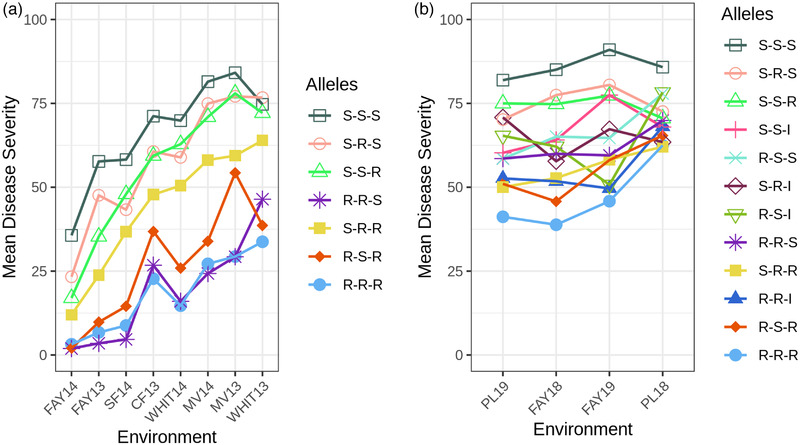
Mean disease severity for each combination of alleles and environments tested for (A) the historic eastern panel and (B) the southeastern elite panel. Disease severity is not directly comparable between panels. The combination of alleles is presented in the format 2AS‐3BS‐4BL. S, susceptibility allele; R, resistance allele; I, intermediate susceptibility–resistance allele. Environments are represented as the combination of location and harvest year. CF, Central Ferry, WA; FAY, Fayetteville, AR; MV, Mount Vernon, WA; PL, Plains, GA; SF, Spitlow agronomy farm, Pullman, WA; WHIT, Whitlow agronomy farm, Pullman, WA

Interaction of multi‐locus genotypes with environments was observed in both panels. A large variation in mean disease severity between environments was observed in the HE panel (Figure 5a), with the two Arkansas environments exhibiting the lowest average SEV scores and the Whitlow Agronomy Farm in 2013 exhibiting the highest average scores. However, there was little crossover‐type interaction of genotypes between environments. The environmental means for the SE panel could not be directly calculated because of the data being transformed into percentile scores. However, the SE panel did display more pronounced crossover‐type interactions of lines in the different genotypic classes between environments than the HE panel (Figure 5b).

### 3.6 Single‐locus genotyping assays

**FIGURE 6:**
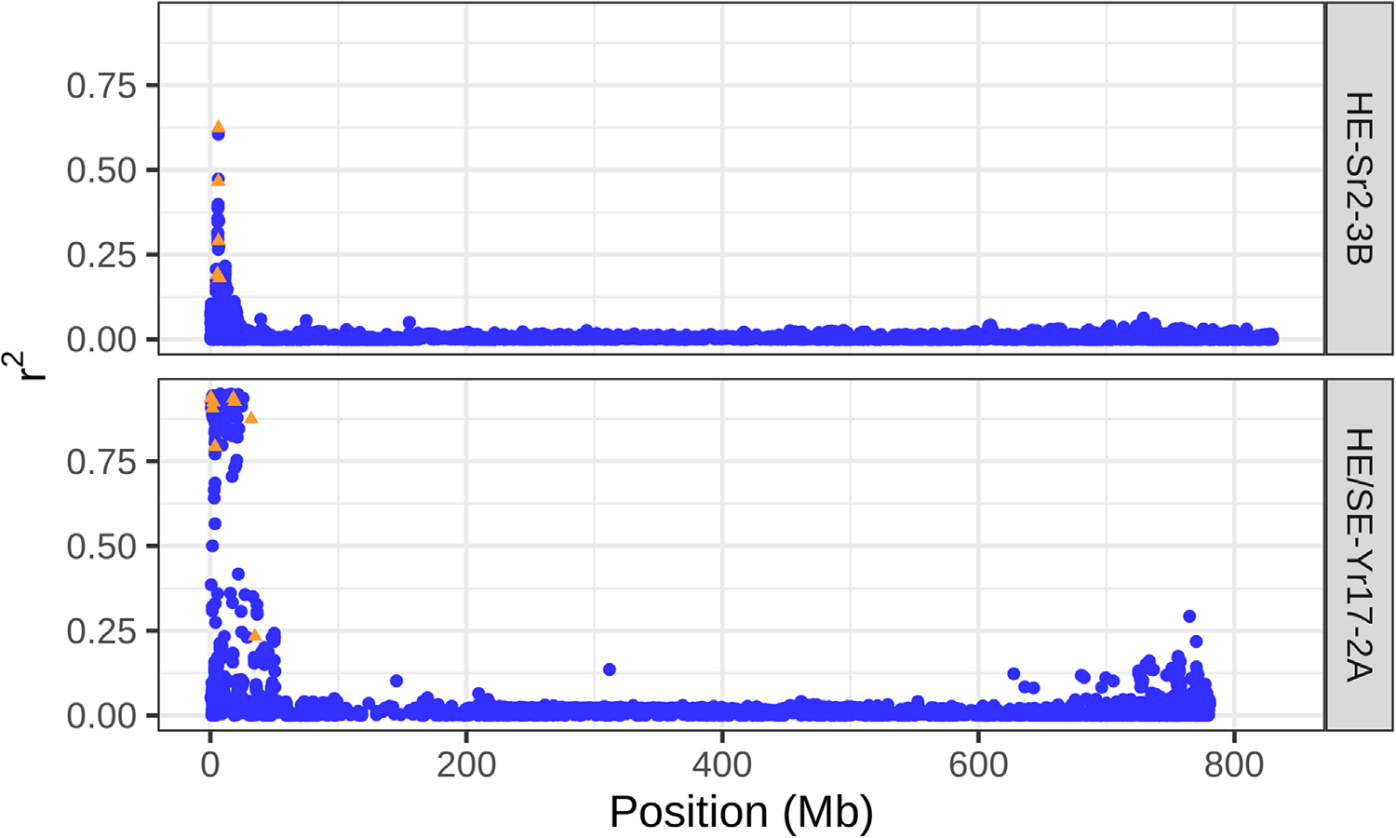
Linkage disequilibrium estimates (*r* ^2^) between all genotyping‐by‐sequencing (GBS) single nucleotide polymorphisms (SNPs) on chromosome 3B and the *Sr2* KASP assay in the historic eastern panel (top panel) and between all GBS SNPs on chromosome 2A and the *Yr17* KASP assay in the historic eastern and southeastern elite panels (bottom panel). SNPs with triangular orange markers were classified as significant in the genome‐wide association analyses

The KASP assay for the *Yr17/Lr37/Sr38* locus, tested in the HE and SE panels, displayed high *r* ^2^ values with SNPs located on chromosome 2A from the distal short end of the chromosome to ∼32 Mb, with peak values of .95 occurring at multiple positions from ∼1.3 to 7.8 Mb (Figure 6). Likewise, the KASP assay for the *Sr2* locus, tested in the HE panel, displayed high *r* ^2^ values with SNPs located on the beginning of chromosome 3B (Figure 6), with a peak value of .62 at 6.38 Mb. No *Sr2*‐positive lines were observed in the assayed portion of the HE panel. The frequency of the negative wild‐type allele (20.5%) was close to the mean minor allele frequency of SNPs within the 3BS QTL region (22.9%), with all other tested lines containing the null allele.

The 3BS KASP markers developed by Liu et al. (2019) were moderately predictive in our germplasm. At the 3BS locus, we observed only a single line containing the resistance haplotype that Liu et al. (2019) describe in the cultivar Skiles (A/G‐G‐G for the markers IWB1836‐IWB20761‐IWA7230). Assuming that other accessions of Skiles could contain one or the other homozygous state for IWB1836, our tested germplasm contained 89 observations of the G‐G‐G haplotype, of which, 95.5% were classified as resistant (Supplemental Table S9A), and 76 observations of the A‐G‐G haplotype, of which only 30.3% were classified as resistant. Finally, our dataset contained 58 observations of the susceptible Avocet‐S A‐A‐A haplotype, of which almost all (98.3%) were categorized as susceptible. The majority of lines (411) contained the haplotype G‐G‐A; of these, nearly all (98.3%) were resistant. The combination of G alleles for both IWB1836 and IWB20761 appeared to be a good but imperfect predictor of resistance at the 3BS locus, as 91.1% of lines with this allelic configuration were classified as resistant.

At the 4BL locus, the validation of our results using Liu et al. (2019) KASP markers yielded more ambiguous results. Our germplasm included 228 lines containing the Skiles resistance haplotype A‐T‐C for the markers IWB33055‐IWB13720‐IWB433 (Supplemental Table S9B). Of these lines, 97.4% were classified as resistant. The Avocet ‘S’ susceptible haplotype C‐G‐T did not appear in our sampled germplasm, though the haplotype C‐G‐C occurred in 309 lines. Of these lines, 68.6% were classified as susceptible, and 13.2% were classified as intermediate susceptible–resistant.

## 4 DISCUSSION

Overall, we found evidence that loci on 2AS, 3BS, and 4BL are driving the majority of stripe rust resistance observed in SRWW germplasm sourced from the southeastern United States. The across‐environment entry mean heritability ranged from .82 for SEV in the SE panel to .94 for IT in the HE panel, while the proportion of across‐environment phenotypic variance explained by all SNPs, or SNP heritability, ranged from .5 for SEV in the SE panel to .62 for IT in the HE panel. As the entry mean heritability calculation is broad‐sense, while the SNP heritability is narrow‐sense, the discrepancy between these two methods of calculating heritability can mostly be attributed to the inclusion of nonadditive effects in the calculation of the former. Missing heritability, or the difference between heritability estimates based on phenotypic measurements and SNP heritability, may additionally explain a portion of the discrepancy. This phenomenon has long been acknowledged in human GWA studies, where individual significant genetic variants often explain only a few percent or fractions of a percent of observed phenotypic variation (Manolio et al., 2009). The missing heritability phenomenon has been less pronounced though still present in plant GWA studies (Brachi, Morris, & Borevitz, 2011). In the present study, estimates of entry mean heritability were consistently higher than their SNP‐based counterparts with differences of .32 for SEV measured in the SE panel and IT measured in the HE panel and .36 for SEV measured in the HE panel. In some cases, such as the SEV ratings in the FAY19 environment of the SE panel, the three major resistance loci explained all the phenotypic variance that could be accounted for by SNPs (Figure 3). However, in most environments, polygenic background resistance accounted for some portion of overall resistance, although the significant MTAs generally explained at least half of the observed variation. The presence of this polygenic effect suggests that additional genetic gains may be realized through the use of genomic selection, either by employing a Bayesian model to allow variable weighting of marker effects to account for the major resistance loci as in Meuwissen, Hayes, and Goddard (2001) or by using a mixed linear model with major resistance loci included as fixed effects as in Sarinelli et al. (2019). However, the usefulness of such an approach depends upon the proportion of phenotypic variance explained by the major resistance loci as the cost of genome‐wide genotyping required for genomic selection is still far higher than the cost of running targeted assays for three individual loci. Below, we describe the three major loci identified in this study in more detail.

### 4.1 The 2NS:2AS translocation

The results of the LD scan using the KASP *Yr17/Lr37/Sr38* assay suggest that the *Ae. ventricosa* 2NS:2AS introgression contains the gene responsible for the resistance effects of the 2AS QTL. This introgression first appears in the tested SRWW germplasm in several University of Georgia accessions developed in the early 2000s. Another candidate resistance gene in this region is the previously mentioned *YrR61*, which was first described in the SRWW cultivar Pioneer 26R61 (Hao et al., 2011). However, Pioneer 26R61 was included in the HE panel and clustered with lines containing the susceptibility haplotype, precluding our consideration of *YrR61* as the causative gene at this locus. In addition, Pioneer 26R61 displayed a relatively susceptible phenotype in the field, with across‐environment SEV and IT ratings of 69.7 and 7, respectively.

Alien introgressions pose some challenges for sequence‐based methods of variant detection. In the present study, two significant SNPs, which appear to belong within the 2NS:2AS alien introgression, aligned to alternative locations in the genome (Figure 2). Both SNPs aligned to chromosome 3D, at positions of 580,937,201 and 580,947,331 bp. This is not unanticipated, as the 2NS:2AS introgression represents a large segment of sequence that is not present in the Chinese Spring reference genome. Failure to align reads to a genomic region in genotypes containing introgressions or deletions may bias short‐read aligners toward selecting alternative alignments as optimum. Alien introgression regions typically contain few mapped reads, and reads that do map to them typically contain high numbers of nucleotides diverging from the reference genome (Cheng et al., 2019). Therefore, alien introgressions typically contain high levels of missing data following SNP calling, and may contain novel alleles that cannot be imputed from the corresponding wheat sequence. Since the Chinese Spring reference genome does not contain the 2NS:2AS translocation, the current reference gene space cannot be mined for candidate resistance genes.

As mentioned previously, recent studies have presented evidence of a HTAP resistance gene in close linkage with *Yr17* (L. Liu et al., 2018; W. Liu et al., 2018; Wan et al., 2016). This configuration of resistance genes could explain some of the historical debate as to whether *Yr17* should be classified as an ASR gene or an APR gene (Milus et al., 2015). Regional stripe rust race screening data (https://striperust.wsu.edu/races/data/) indicates that in the year 2013, 158 of 204 total *P. striiformis* isolates collected in Washington (77.4%) were *Yr17* virulent, while 100% of the 24 isolates collected in Arkansas were *Yr17* virulent. In the year 2014, 96 of 156 total isolates collected in Washington (61.5%) were *Yr17* virulent. State‐level data is not available for Arkansas in 2014 but in the region encompassing Arkansas (Region 7), 100% of 12 collected isolates were *Yr17* virulent. In 2018, all seven isolates collected in Georgia and all seven collected in Region 7 were *Yr17* virulent. In 2019, all 14 Region 7 isolates were *Yr17* virulent, while all three isolates from the region encompassing Georgia (Region 10) were *Yr17* virulent. Thus, the only environments containing *Yr17*‐avirulent races in the study were the northwestern environments of the HE panel, though it should be noted that both the SF14 and WHIT14 environments received some artificial inoculation with *Yr17*‐virulent isolates.

The finding that 2NS:2AS generally explains a larger portion of variance for SEV in the HE panel than in the SE panel (Figure 3) supports the hypothesis of a HTAP gene tightly linked with *Yr17*. The HTAP gene produces a more quantitative resistance against all *P. striiformis* races (L. Liu et al., 2018) than the race‐specific, more qualitative resistance of *Yr17*. The quantitative action of the HTAP gene in combination with *Yr17* virulence in the pathogen populations in Georgia and Arkansas may explain the lower proportion of phenotypic variance explained by the 2AS locus in the SE panel. It should also be noted that we may expect the HTAP gene to confer stronger, more consistent resistance in southeastern environments, which experience shorter, warmer winter wheat growing seasons than northwestern environments. Despite its lower contribution to stripe rust resistance in more recent testing, the frequency of the 2NS:2AS translocation increased from 11% in the HE panel to 48% in the SE panel. However, we cannot currently determine whether this sharp increase in frequency is due to direct selection for stripe rust resistance, indirect selection resulting from selection for the linked *Lr37* or *Sr38* genes, or simply is due to the dissemination of parental lines carrying the translocation following its introduction to regional germplasm in the early 2000s.

### 4.2 The 3BS resistance QTL

Of the three major QTL responsible for the majority of stripe rust resistance in this study, the 3BS QTL was physically the smallest, with MTAs in both panels ranging from 5,580,613 to 7,031,798 bp. Several named stripe rust resistance genes have been localized to the distal portion of the short arm of chromosome 3B. However, because most previous QTL mapping and GWA studies have not had access to a reference genome or physically anchored markers, comparisons with our results is difficult. *Yr30* is an APR gene introgressed from emmer wheat [*Triticum turgidum* L. subsp. *dicoccon* (Schrank) Thell.] cv. Yaroslav. *Yr30* was introgressed with stem rust resistance gene *Sr2*, from the cross Yaroslav emmer × ‘Marquis’ (McFadden, 1930), producing the bread wheat cultivar Hope (Spielmeyer et al., 2003). The linked *Sr2* gene has historically been difficult to phenotype in the field, as it is recessive and only offers partial resistance against most stem rust races (Mago et al., 2011). However, the pseudo black‐chaff trait has long been used to select for *Sr2*, as it causes conspicuous dark pigmentation on stem internodes and glumes in the field and is in tight linkage with *Sr2* (Kota et al., 2006).

No *Sr2*‐positive lines were detected among the 254 lines from the HE panel assayed with the Sr2 KASP marker. This result is as expected, as lines containing *Sr2* are known to be exceedingly rare in SRWW germplasm (https://www.ars.usda.gov/southeast-area/raleigh-nc/plant-science-research/docs/small-grains-genotyping-laboratory/compendiums/). The maximum LD between the Sr2 KASP assay and GBS SNPs on 3BS (*r* ^2^ = .62) was not as strong as the LD between the *Yr17* KASP assay and GBS SNPs on 2AS. This suggests that the *Sr2* assay may not be accurately resolving the molecular variation within the 3BS region. This finding, in combination with the lack of *Sr2*‐positive lines in the HE panel, suggests that *Sr2*/*Yr30* is collocated with the 3BS QTL but is not the gene responsible for the resistance being observed. Instead, one or more alternate resistance genes in this region are likely responsible for the observed phenotypic variation.

Several other resistance genes have previously been reported in the same region. These include the ASR gene *Yr57*, which was first described in the cross of an Australian landrace, AUS27858, with ‘Westonia’ (Randhawa, Bariana, Mago, & Bansal, 2015). Within this population, *Yr57* mapped to a region flanked by the markers *gwm389* and *BS00062676* with genetic positions of 2.0 and 2.3 cM, respectively. *Yr57* does not appear to be a likely candidate for the 3BS QTL in the current study, as it was first described in unadapted source germplasm only recently. The ASR gene *Yr4* (syn. *YrRub*) was mapped to 3BS in the Australian spring wheat cultivar Rubric (AUS33333) in progeny of the cross Rubric × Avocet ‘S’ and is present in the cultivars Hybrid 46 and Avalon (Bansal, Hayden, Gill, & Bariana, 2010). The SSR marker *cfb3530* was found to be closely linked to *Yr4* at a distance of 2.9 ± 1.3 cM. The resistance gene *Yr58* was identified in a doubled‐haploid population derived from resistant Indian spring wheat landrace W195 crossed with susceptible South Australian cultivar BTSS, in which it interacted with *Yr46* on chromosome 4D to offer enhanced resistance (Chhetri, Bariana, Kandiah, & Bansal, 2016). Marker positions for *Yr58* were defined using coordinates from the earlier contig‐level Wheat Chromosome Survey Sequence. However, using BLAST on the primer sequences of the most diagnostic markers of Chhetri et al. (2016) revealed positions of ∼2.2 and ∼3.2 Mb for the SSR markers *sun476* and *sun533*, respectively, and a position of ∼3.6 Mb for diversity arrays technology sequencing marker 3023704. This places *Yr58* slightly distal to our 3BS QTL, though likely close enough that its consideration should not be ruled out. Finally, the gene given temporary designation *Yrns‐B1* was mapped to the distal end of 3BS in the line Lgst. 79‐74 in a cross with the susceptible line ‘Winzi’ (Börner, Röder, Unger, & Meinel, 2000). *Yrns‐B1* was located 20.5 and 21.7 cM proximal to the microsatellite marker *Xgwm493* in two separate years of stripe rust screening data respectively. This position also places it proximal to our 3BS marker.

Quantitative trait loci on 3BS have also been reported in recent biparental QTL mapping studies. Subramanian et al. (2016) performed mapping in southeastern U.S. SRWW germplasm using recombinant inbred lines (RILs) derived from the cross ‘Coker 9835’ × VA96W‐270. The authors reported the QTL *QYr.ar.3BS* explaining between 14 and 31% of phenotypic variation depending on the combination of environment and trait (either SEV or area under the disease progression curve). *QYr.ar.3BS* was located from approximately 0 to 22 cM with a peak logarithm of the odds score occurring at 3.0 or 6.3 cM. Liu et al. (2019) also mapped a QTL to 3BS, *QYrsk.wgp‐3BS*, using a population of 129 doubled‐haploid lines developed from the cross of soft white winter wheat ‘Skiles’ with spring wheat line Avocet ‘S’. This locus explained between 10.2 and 28.2% of phenotypic variation, depending on the combination of environment and measured trait (one of IT, SEV, or relative area under the disease progression curve). By aligning SNP and SSR markers with the v1.0 Chinese Spring reference sequence, they determined that *QYrsk.wgp‐3BS* was located between 2.4 and 18.2 Mb on chromosome 3B.

Using interpolated genetic positions from the Opata × Synthetic W7984 population described in Gutierrez‐Gonzalez, Mascher, Poland, and Muehlbauer (2019) places our QTL between 0 and 4.82 cM when using the across‐environment BLUEs as phenotypes. This locates it within the same region as the QTL reported by Subramanian et al. (2016) and slightly proximal to the position for *Yr57* mapped by Randhawa et al. (2015). However, the genetic positions derived from the Opata × Synthetic W7984 population should be treated as approximations in the HE and SE panels. Our QTL region is also encompassed within the 3BS QTL region identified by Liu et al. (2019). The comparison of our results with those of Liu et al. (2019) is difficult because of the presence of a heterozygous A/G call for the marker IWB1836 in the Skiles accession that they tested. While the G‐G‐G haplotype for IWB1836, IWB20761, and IWA7230 was reasonably predictive of resistance in our material, the A‐G‐G haplotype was not. In addition, the G‐G‐A haplotype was most prevalent in our germplasm, where it was associated with a resistant phenotype, though this haplotype was relatively rare in Liu et al. (2019). For these reasons, we cannot at this time determine whether our QTL on 3BS is the *QYrsk.wgp‐3BS* QTL described by Liu et al. (2019).

### 4.3 The 4BL resistance QTL

The QTL detected on 4BL in the present study was physically large, spanning a region from approximately 530 to 599 Mb (Figure 2). The unstable effects of this QTL across the two panels is concerning in regards to its widespread deployment in SRWW germplasm. In our study, only the 4BL_2 haplotype provided consistent resistance across both testing panels, with the 4BL_4 haplotype offering at least intermediate resistance in both panels. We attribute this to the significant epistatic 3BS:4BL interactions in the SE panel, as opposed to the purely additive effects of both loci observed in the HE panel. Nevertheless, the large effects of the 4BL QTL in the newer germplasm of the SE panel make it worth pursuing as a source of stripe rust resistance.

In the current study, no KASP data was available to test for the presence of known resistance genes at this locus, though several named stripe rust resistance genes have been reported on the distal region of 4BL. *Yr50* is an ASR gene introgressed into hexaploid wheat from intermediate wheatgrass (*Thinopyrum intermedium* (Host) Barkworth & D. R. Dewey subsp. *intermedium*) flanked by the markers *Xbarc1096* and *Xwmc47* (Liu et al., 2013). However, this introgression was carried out too recently for *Yr50* to be relevant to the germplasm tested in the present study. *Yr62* is an HTAP gene mapped to 4BL in the Portuguese spring wheat accession PI 192252 from a cross with the susceptible parent Avocet ‘S’ (Lu et al., 2014). *Yr62* is flanked by the markers *Xgwm251* and *Xgwm192*, placing it between 41.9 and 44.5 cM. It was also found to be in partial linkage with *Yr50*, with the two being separated by a distance of 27.1 ± 8.6 cM. PI 192252 was deposited into the USDA–ARS National Small Grains Collection in 1950, and *Yr62*’s location is in the same vicinity as the significant markers in our study (∼50 cM). Therefore, while it is unlikely that the 4BL resistance QTL observed in the present study is *Yr62*, this possibility cannot be ruled out. Recently, *YrZH22* was mapped on 4BL by performing bulked segregant RNA sequencing on a population of RILs derived from the cross of two Chinese lines: ‘Zhoumai 22’ × ‘Mingxian 169’ (Wang et al., 2017). Flanking markers for this QTL, *WGGB133* and *WGGB146*, align to the Chinese Spring v1.0 reference genome chromosome 4B at positions of approximately 581 and 672 Mb, respectively. This positions *YrZH22* as overlapping slightly with the 4BL QTL identified in the present study.

Two recent QTL mapping studies using southeastern U.S. winter wheat germplasm have detected a 4BL resistance QTL. In a biparental population of RILs formed from the cross ‘USG 3555’ × ‘NC‐Neuse’, Christopher et al. (2013) found the consistently large‐effect resistance QTL *QYrus.vt‐4BL* in USG 3555, explaining between 66.1 and 80% of variation for SEV and between 62.1 and 82.5% of variation for IT, depending upon environment. However, the confidence interval for this QTL spanned nearly the entire chromosome arm. In the present study, USG 3555 contained the 4BL_2 haplotype, which was classified as a resistance haplotype in both panels, while NC‐Neuse contained the 4BL_1 haplotype, which was classified as a susceptibility haplotype in both panels. In addition, the previously mentioned Coker 9835 × VA96W‐270 population QTL mapping study by Subramanian et al. (2016) detected the QTL *QYr.ar‐4BL* for stripe rust disease severity on 4BL. This locus explained between 17 and 26.3% of variation for SEV ratings, and its location was narrowed to a region spanning approximately 49 to 65 cM, with peak logarithm of the odds scores occurring between 57.5 and 64.6 cM. The resistant parent VA96W‐270 from Subramanian et al. (2016) was not included in the present study; however, the susceptible parent, Coker 9835, was included and exhibited the expected 4BL_1 susceptibility haplotype.

Two other recent mapping studies are of note. Liu et al. (2019) mapped a resistance QTL, *QYrsk.wgp‐4BL*, to 4BL in the Skiles × Avocet ‘S’ doubled‐haploid population. This locus explained from 17.3 to 41.8% of disease resistance, depending upon the environment in combination with the rated phenotypic trait. They give marker physical positions in terms of the long arm rather than the entire 4B chromosome. However, a BLAST search using their three diagnostic markers for *QYrsk.wgp‐4BL* indicates that they align to positions on chromosome 4B ranging from approximately 526 to 567 Mb, an interval which is encompassed within our 4BL QTL region. As previously mentioned, our validation using KASP markers of Liu et al. (2019) yielded inconclusive results. This may be due to the presence of an additional intermediate resistance–susceptibility phenotypic category in our material. Therefore we cannot definitively state whether or not we have detected *QYrsk.wgp‐4BL* in our germplasm.

Another QTL, *QYr.nwafu‐4BL*, was mapped to 4BL using RILs derived from the cross Mingxian 169 × P10103, and was verified in the cross ‘Zhengmai 9023’ × P10103 (Wu et al., 2018). This was a large effect QTL explaining between 30.5 and 42% of phenotypic variation for IT and SEV, depending upon the trait/environment combination. *QYr.nwafu‐4BL* was located at approximately 90 cM on chromosome 4B, a location that places it approximately 40 cM distal from our 4BL QTL.

### 4.4 Additional minor QTLs

As previously noted, in addition to the three major QTL located on 2AS, 3BS, and 4BL, several additional potential QTL were identified. One MTA affecting the trait SEV in the MV13 environment of the HE panel was located at 2A:34,731,966 bp. Although this may place it within the 2NS:2AS translocation, it exhibits relatively weak LD with all other MTAs in the translocation, which all exhibit very high LD among one another (Figure 2). Therefore, this MTA may represent a separate locus that is proximal to 2NS:2AS. It does not appear that this MTA represents the previously mentioned *YrR61* resistance gene, as Pioneer 26R61 contains the unfavorable major allele at this position. One additional MTA was located on chromosome 2B at ∼88 Mb. This locus affected the trait SEV in the environment FAY13 in the HE panel. The short arm of chromosome 2B contains a large number of previously identified resistance genes; for a recent review addressing them, we refer readers to Wang and Chen (2017).

### 4.5 Allelic combinations

There was a wide variety of allelic combinations between the three major QTL regions (Figure 4). The combination of the wild‐type 2AS locus with resistance alleles at the 3BS and 4BL loci was the most common in both panels. This combination, which we label the S‐R‐R allelic configuration, predates the establishment of modern breeding techniques, as it is present in Goens and Mediterranean, the two oldest lines that we tested. However, an important distinction between these two is that Goens contains the more stable 4BL_2 resistance haplotype, while Mediterranean contains the 4BL_4 haplotype, which functioned as a resistance allele in the HE panel and as an intermediate resistance–susceptibility allele in the SE panel. The SE panel exhibited more crossover‐type genotype‐by‐environment interaction than the HE panel (Figure 5). This was surprising, as the HE panel was tested in both southeastern and Pacific Northwest environments as opposed to the SE panel, which was only tested in the southeast. In addition, the HE panel was more diverse overall, including four mainly regional subpopulations (Figure 1), which initially led us to assume that it would exhibit greater genotype‐by‐environment interaction effects. The SE panel contained a total of 12 allelic combinations of the three major QTLs compared with seven present in the HE panel. This greater allelic diversity may simply be due to the larger size of the SE panel. However, it could also represent the creation of new combinations in modern breeding lines. In the HE panel, lines containing the 2NS:2AS translocation consistently displayed the highest disease resistance across environments. Of the lines lacking 2NS:2AS, those with the S‐R‐R combination consistently had the highest resistance across environments. In the SE panel, the S‐R‐R combination offered relatively stable performance across environments and outperformed several allelic combinations containing the 2NS:2AS translocation. The S‐R‐R combination was only consistently outperformed by lines containing resistance haplotypes at all three loci, and its performance was mostly on par with the allelic combination consisting of resistance haplotypes at 2AS and 3BS with an intermediate resistance haplotype at 4BL (R‐R‐I) and the combination consisting of resistance haplotypes at 2AS and 4BL (R‐S‐R). In both cases the S‐R‐R combination only clearly underperformed these other two combinations in a single environment. It should be noted that in the SE panel the S‐R‐R allelic combination only includes lines containing the 4BL_2 haplotype, while in the HE panel, it includes lines with any 4BL haplotype other than 4BL_1.

## 5 CONCLUSION

Taken as a whole, the data suggest that the 4BL QTL plays a large role in adult plant stripe rust resistance in the contemporary SRWW breeding germplasm of the southeastern United States, with the 3BS QTL and *Ae. ventricosa* 2NS:2AS translocation providing additional resistance. Different haplotypes in the 4BL QTL region exhibited unstable effects across the two testing panels and were involved in epistatic interactions in the SE panel. These unstable effects may be indicative of different resistance gene alleles, variability in the pathogen population, or environmental sensitivity of resistance. However, the 4BL_2 haplotype provided consistent resistance across both testing panels and subsets of environments. The adoption of the 2NS:2AS translocation into regional germplasm proceeded rapidly following its initial introgression into University of Georgia breeding material in the early 2000s despite its less important role in providing stripe rust resistance in more modern germplasm. The findings of the present study suggest that *Yr17* resistance has been overcome in the southeast United States for some time. Nevertheless, the presence of the linked HTAP gene on the translocation still confers adult plant resistance and likely accounts for the majority of 2AS resistance observed in contemporary SRWW breeding material in many environments, though this hypothesis requires further empirical verification. The causative genes underlying the 3BS and 4BL QTL remain unknown. The increasing availability of hexaploid wheat assemblies and long‐read sequencing technology may soon allow for the possibility of identifying the causal genes underlying these loci through comparative genomics, eliminating the need to develop fine mapping populations.

## AUTHOR CONTRIBUTIONS

Keith R Merrill: Conceptualization; Data curation; Formal analysis; Investigation; Methodology; Resources; Writing‐review & editing. Peter Bulli: Investigation; Resources. Richard Esten Mason: Investigation; Resources; Supervision; Writing‐review & editing. Mohamed Mergoum: Investigation; Resources; Supervision; Writing‐review & editing. Jerry Johnson: Investigation; Resources; Supervision; Writing‐review & editing. Suraj Sapkota: Investigation; Resources; Writing‐review & editing. Benjamin Lopez: Investigation; Resources. Gina Brown‐Guedira: Conceptualization; Funding acquisition; Methodology; Project administration; Resources; Supervision; Writing‐review & editing

## CONFLICT OF INTEREST

The authors declare that they have no conflict of interest.

## Supporting information

Supplemental Figure S1: Beagle imputation accuracy.
Supplemental Figure S2: ADMIXTURE subpopulation classification error for 5‐fold cross‐validation using the Historical Eastern Panel (HEP) and Southeastern Elite Panel (SEP), for number of subpopulations (k) ranging from one to ten.
Supplemental Figure S3: Biplot of first two principal components of the full, unimputed genotypic dataset, thinned based on pairwise linkage disequilibrium such that all SNPs are in approximate linkage equilibrium.
Supplemental Figure S4: Density plot of discriminant analysis of principal components (DAPC) clustering for all genotypes in the HE and SE panels calculated using all SNPs falling within the 2AS resistance QTL (i.e. the *Ae. ventricosa* 2NS:2AS translocation).
Supplemental Figure S5: Scatter plot of discriminant analysis of principal components (DAPC) clustering for all genotypes in the HE and SE panels calculated using all SNPs falling within the 3BS resistance QTL.
Supplemental Figure S6: Scatter plot of discriminant analysis of principal components (DAPC) clustering for all genotypes in the HE and SE panels calculated using all SNPs falling within the 4BL resistance QTL.
Supplemental Figure S7: Interaction plot for the 2AS and 3BS QTLs in the SE panel across different 4BL haplotypes.
Supplemental Figure S8: Interaction plot for the 3BS and 4BL QTLs in the SE panel across different 2AS haplotypes

Supplemental Table S1: Primers used for KASP *Yr17/Lr37/Sr38* and *Sr2* assays
Supplemental Table S2: 3BS and 4BL QTL KASP markers developed by Liu et al., 2019
(https://doi.org/10.1007/s00122‐019‐03307‐2) tested in 685 soft red winter wheat accessions
Supplemental Table S3: Across‐environment variances and heritability estimates for all tested trait/panel combinations
Supplemental Table S4: Genome‐based restricted maximum likelihood (GREML) estimates of phenotypic variance explained by all SNPs jointly
Supplemental Table S5: Summary of ANOVA model for three major loci, three two‐way epistatic interactions, and one three‐way epistatic interaction in the Historical Eastern (HE) panel
Supplemental Table S6: Summary of ANOVA model for three major loci, three two‐way epistatic interactions, and one three‐way epistatic interaction in the Southeastern Elite (SE) panel
Supplemental Table S7: Across‐environment disease severity (SEV), haplotypes and corresponding resistance classification of the 2AS, 3BS, and 4BL QTLs, and allelic combination of all three QTLs for each genotype in the HE panel.
Supplemental Table S8: Across‐environment disease severity (SEV), haplotypes and corresponding resistance classification of the 2AS, 3BS, and 4BL QTLs, and allelic combination of all three QTLs for each genotype in the SE panel.
Supplemental Table S9‐A: Contingency table of 3BS haplotypes defined by three KASP markers from Liu et al., 2019 (https://doi.org/10.1007/s00122‐019‐03307‐2) vs. GBS marker haplotype categories derived in present study. Haplotypes containing heterozygous KASP marker calls have been omitted.

## Data Availability

The genotypic, phenotypic, and covariate KASP marker data used for the GWA analyses are deposited on FigShare (https://doi.org/10.6084/m9.figshare.9920096).
