# Supplementary material for: Analysis of the primary sources of quantitative adult plant resistance to stripe rust in U.S. soft red winter wheat germplasm
Source: Plant Genome. 2021 Feb 17;14(1):e20082. doi: 10.1002/tpg2.20082 (PMC12807305; doi:10.1002/tpg2.20082)
Supplement: Supplementary file 1 [file TPG2-14-e20082-s002.docx]

**Analysis of the Primary Sources of Quantitative Adult Plant Resistance to Stripe Rust in U.S. Soft Red Winter Wheat Germplasm**

Brian P. Ward, Keith Merrill, Peter Bulli, Mike Pumphrey, Richard Esten Mason, Jerry Johnson, Mohamed Mergoum, Suraj Sapkota, Benjamin Lopez, Gina Brown-Guedira

**Supplemental Figures**

**Supplemental Figure S1:** Beagle imputation accuracy. 10,000 randomly-selected non-missing genotype calls from the genotypic dataset were masked and imputed with Beagle 5. Imputed calls were then compared against actual calls. SNPs were binned by their proportion of missing data and minor allele frequency (MAF).

**Supplemental Figure S2**: ADMIXTURE subpopulation classification error for 5-fold cross-validation using the Historical Eastern Panel (HEP) and Southeastern Elite Panel (SEP), for number of subpopulations (k) ranging from one to ten.

**Supplemental Figure S3:** Biplot of first two principal components of the full, unimputed genotypic dataset, thinned based on pairwise linkage disequilibrium such that all SNPs are in approximate linkage equilibrium. The dataset contains lines belonging to the four subpopulations of the Historical Eastern Panel (HEP), as well as lines of the Southeastern Elite Panel (SEP), which did not exhibit any significant population structure.

**Supplemental Figure S4**: Density plot of discriminant analysis of principal components (DAPC) clustering for all genotypes in the HE and SE panels calculated using all SNPs falling within the 2AS resistance QTL (i.e. the *Ae. ventricosa* 2NS:2AS translocation).

**Supplemental Figure S5**: Scatter plot of discriminant analysis of principal components (DAPC) clustering for all genotypes in the HE and SE panels calculated using all SNPs falling within the 3BS resistance QTL.

**Supplemental Figure S6**: Scatter plot of discriminant analysis of principal components (DAPC) clustering for all genotypes in the HE and SE panels calculated using all SNPs falling within the 4BL resistance QTL.

**Supplemental Figure S7**: Interaction plot for the 2AS and 3BS QTLs in the SE panel across different 4BL haplotypes.

WT wild-type (susceptibility) allele; 2NS:2AS *Ae. ventricosa* alien introgression (resistance) allele; 3BS_1 3BS susceptibility allele, 3BS_2, 3BS_3 3BS resistance alleles; 4BL_1, 4BL_3 4BL susceptibility alleles; 4BL_2 4BL resistance allele; 4BL_4 4BL intermediate susceptibility/resistance allele

**Supplemental Figure S8**: Interaction plot for the 3BS and 4BL QTLs in the SE panel across different 2AS haplotypes

WT wild-type (susceptibility) allele; 2NS:2AS *Ae. ventricosa* alien introgression (resistance) allele; 3BS_1 3BS susceptibility allele, 3BS_2, 3BS_3 3BS resistance alleles; 4BL_1, 4BL_3 4BL susceptibility alleles; 4BL_2 4BL resistance allele; 4BL_4 4BL intermediate susceptibility/resistance allele
